# Recent Advances in and Applications of Electrochemical Sensors Based on Covalent Organic Frameworks for Food Safety Analysis

**DOI:** 10.3390/foods12234274

**Published:** 2023-11-27

**Authors:** Hongwei Zhu, Minjie Li, Cuilin Cheng, Ying Han, Shiyao Fu, Ruiling Li, Gaofeng Cao, Miaomiao Liu, Can Cui, Jia Liu, Xin Yang

**Affiliations:** 1 Beijing Key Laboratory of Nutrition & Health and Food Safety, Beijing Engineering Laboratory of Geriatric Nutrition & Foods, COFCO Nutrition and Health Research Institute Co., Ltd., Beijing 102209, China; zhuhongwei1994@126.com (H.Z.);liminjie@cofco.com (M.L.); cuican@cofco.com (C.C.); 2 School of Medicine and Health, Harbin Institute of Technology, Harbin 150001, China; ccuilin@hit.edu.cn (C.C.); hany1994hit@163.com (Y.H.); 15164510974@163.com (S.F.); lirl1995@163.com (R.L.); 3 Internal Trade Food Science Research Institute Co., Ltd., Beijing 102209, China; 4 School of Chemistry and Chemical Engineering, Harbin Institute of Technology, Harbin 150001, China; 5 COFCO Corporation, Beijing 100020, China; caogf@cofco.com (G.C.); liumiaomiao@cofco.com (M.L.)

**Keywords:** covalent organic frameworks, electrochemical sensors, food safety, pesticides, antibiotics

## Abstract

The international community has been paying close attention to the issue of food safety as a matter of public health. The presence of a wide range of contaminants in food poses a significant threat to human health, making it vital to develop detection methods for monitoring these chemical contaminants. Electrochemical sensors using emerging materials have been widely employed to detect food-derived contaminants. Covalent organic frameworks (COFs) have the potential for extensive applications due to their unique structure, high surface area, and tunable pore sizes. The review summarizes and explores recent advances in electrochemical sensors modified with COFs for detecting pesticides, antibiotics, heavy metal ions, and other food contaminants. Furthermore, future challenges and possible solutions will be discussed regarding food safety analysis using COFs.

## 1. Introduction

The safety of food is of vital importance to the health of people and to the long-term stability of society in general [1]. Food forms the basis of human survival and is essential for maintaining a stable and sustained existence [2]. Food safety is defined by the Food Safety Law of the People’s Republic of China as non-toxic, harmless, and meeting the nutritional requirements without causing acute, subacute, or chronic harm to humans. It is noteworthy that the European Union, the United States, and other countries have very similar definitions of food safety, even if they express it in a slightly different manner.

Globally, the top 100 food and beverage companies generated revenues of USD 1.3 trillion in 2019, equivalent to approximately CNY 9.2 trillion [3]. However, with the achievement of economic globalization, food safety issues have become a worldwide issue that has impacted more than just an individual country or region. Currently, food safety is subjected to many challenges due to differences in the natural environment in different countries and regions. It is possible for food to become unavoidably contaminated during its preparation, transportation, and storage, regardless of how rigorous and meticulous the handling procedures are. As a result of the excessive use of veterinary drugs and pesticides [4] and heavy metal ions [5] and the introduction of illegal additives [6], in particular, food can be contaminated in a variety of ways throughout the food chain [7]. In addition to these factors, hazardous food contaminants deserve special attention because even in low concentrations, they are able to cause serious diseases such as cancer [8], and furthermore, fungi that contaminate food such as aspergillus, penicillium, and neotyphodium [9] pose a serious threat to human health and safety. The food safety industry has experienced some extremely detrimental incidents in recent years, including the melamine incident at Sanlu Group in China in 2008, the salmonella-contaminated peanut butter incident at Peanut Corporation of America from 2008 to 2009, the E. coli contamination of bean sprouts in the European Union in 2011, and the contamination of milk powder in 2013 with Clostridium botulinum toxin by Fonterra in New Zealand. Due to these recent food safety incidents, the global society has been paying close attention to this issue, and many countries and regions have adjusted their policies and intensified their supervision on food regulation [10]. In addition to posing significant risks to the health and safety of the general public, these frequent food safety incidents also cause significant losses for the industries that are directly affected by the incidents.

Having a rational and effective approach for food testing is an essential component of food safety management. Conducting well-informed research on testing techniques can provide powerful assurances regarding food safety being maintained continuously.

### 1.1. Electrochemical Sensors and Their Role in Food Safety Analysis

The foundation of any food safety program is improved food safety testing techniques, which are key in addressing food safety. Food safety testing methods can be classified as traditional or rapid detection. Usually, conventional methods consist of using techniques such as gas chromatography–mass spectrometry [11], high-performance liquid chromatography [12], and liquid chromatography–mass spectrometry [13,14] to determine the identity of food. These methods are usually performed in laboratories with sophisticated equipment. They are frequently used as reference standards to ensure food safety because of their high sensitivity, accuracy, precision, and repeatability. However, the length of their analysis cycle and their low throughput are their limitations. Rapid detection methods, on the other hand, deliver faster results. It is common to use these methods of qualitative or semi-quantitative screening of target analytes [15]. The benefits of electrochemical detection methods are many, including affordability, simplicity, ease of operation, miniaturization, and diversification, over traditional methods such as spectroscopy and chromatography [16]. In addition, electrochemical detection is suitable for automated control as well as online sensitive and rapid analysis since it can be conducted remotely [17]. They can be applied to biomedical sciences, pharmaceuticals, environmental sciences, and food sciences, and are considered to be one of the most dynamic and promising analytical techniques [18].

**Figure 1:**
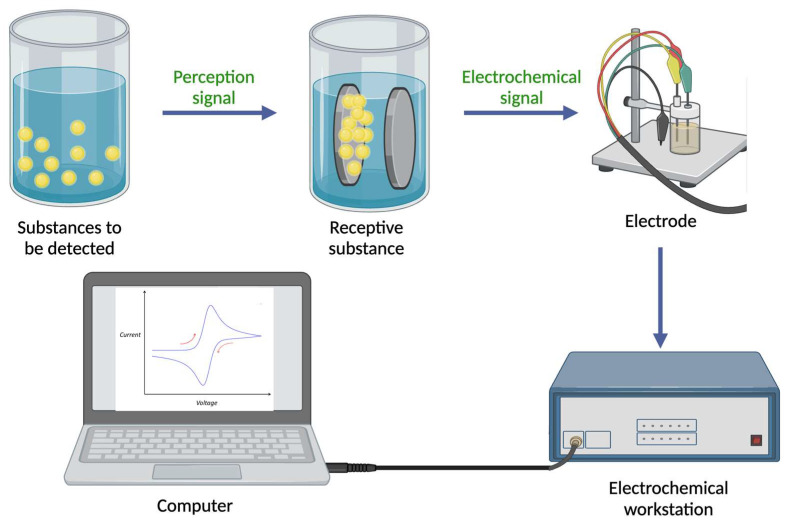
Diagram of an electrochemical sensor. (The mechanism map was created with BioRender.com.)

The primary objective of electrochemical detection techniques is to qualitatively or quantitatively analyze and measure target substances based on their electrical and electrochemical properties through the use of electrochemical sensors [19]. There are two main components of electrochemical sensors: a molecular recognition system and a system for converting information into electrical signals (the principle of electrochemical sensors is illustrated in Figure 1). Based on the measured chemical parameters, response signals are generated in the form of voltages, currents, or changes in light intensity. These signals are then amplified, converted, and finally transformed into analyzable signals that indicate the amount of target analyte present in the sample using electronic systems [20]. It is widely known that sensors with electrochemical integration are widely used in a variety of fields, including industry, transportation, environmental monitoring, and medical surveillance. Sensors based on electrochemical reactions play an important role in combining sensing technology and electrochemical analysis technology. Electrochemical sensors have been widely applied and developed since the 1960s, with electrodes serving as the basic component [21]. The electrodes play an integral role in the overall performance of electrochemical sensors due to their functionality and interfacial performance. However, one of the main challenges is making electrodes more responsive and selective to desired reactions. Nanotechnology has made rapid progress since the 1980s, resulting in many nanomaterials with exceptional performance and unique structures, and these materials have excellent biocompatibility, a high surface chemical activity, a large specific surface area, and a high electron transfer efficiency, thus facilitating the use of nanomaterials (for instance, COF [22,23,24], MOF [25,26], MIP [27,28,29], among others [30,31]) in electrochemical sensing). New types of electrochemical sensors have been developed as a result of the convergence of nanotechnology and sensing technology, which has attracted increasing attention. In addition to providing the rapid identification of basic food components, electrochemical sensors are capable of detecting harmful substances such as heavy metal ions [32], foodborne pathogens [33], pesticide residues [34], and food additives [35].

### 1.2. Covalent Organic Frameworks (COFs) and Their Potential Applications in Sensor Technology

As a general rule, nanomaterials are materials with at least one dimension out of the three dimensions within the nanometer size range (1–100 nm), or they are formed from basic constituents with such dimensions. Nanomaterials have unique physicochemical properties in optics, electronics, magnetism, heat, mechanics, and other fields as a result of their unique surface effects, small size effects, quantum effects, and macroscopic quantum tunneling effects [36]. The use of these technologies is widespread in fields such as food technology, electronics manufacturing, chemical engineering, and many others [37,38,39]. Moreover, nanomaterials have a small size effect, leading to a large specific surface area and a high surface energy, and they have abundant surface-active sites and exhibit an ease of functionalization. This contributes to a high catalytic efficiency as well as an excellent biocompatibility, greatly enhancing their potential of being electrochemically research [40,41]. Furthermore, nanocomposites are composed of materials in which nanoparticles are uniformly dispersed within the matrix material. It is important to note that unlike traditional single-phase nanomaterials, nanocomposites can consist of a combination of metal nanoparticles with resins or gels, polymer materials, porous inorganic materials, porous organic materials, and various types of metal nanoparticles. There have been several developments in nanomaterials so far, including carbon materials (graphene, carbon nanotubes, carbon foam, carbon fibers, carbon spheres, porous carbon materials, etc.), metal–organic frameworks (MOFs), zeolitic imidazolate frameworks (ZIFs), covalent organic frameworks (COFs), among others [42,43,44,45].

The covalent organic frameworks (COFs) represent a new class of organic porous materials [46]. In 2005, Yaghi and colleagues were successful in synthesizing two-dimensional COFs, COF-1 and COF-5, via the condensation reaction between phenylboronic acid and 2,3,6,7,10,11-hexahydroxytriphenylene. These COFs with high surface areas (711 and 1590 m^2^ g^−1^, respectively), high thermal stability, and permanent porosity were compared [47]. Following this, Yaghi proposed three-dimensional COFs in 2007, including COF-102, COF-105, and COF-108 [48]. There has been a great deal of interest in COFs since their introduction, and they have been applied in a variety of fields. As a result of their flexible polygonal frameworks that are easy to design and control, COFs have been widely applied for a variety of purposes, such as catalysis, energy storage, water treatment, drug delivery, among others [49]. Compared to conventional electronic components, COF and overoxidized PEDOT or PEDOT/PSS have better electrical signal transduction. Controlling the electrical properties of overoxidized PEDOT and PEDOT/PSS is the primary method of actuating the device. A change in the electrical conductivity of PEDOT/PSS can be achieved by applying an electric field or conducting an electrochemical reaction, thus allowing the material to be controlled in terms of its properties and functions [50]. In contrast, the COF undergoes physical or chemical changes through the adsorption or desorption of internal molecules, which cause changes in the signaling pathway. It is estimated that over 10,000 papers (from WOS) have been published over the past five years due to the wide potential applications of this unique material across many fields.

## 2. Fundamentals of COF-Based Electrochemical Sensors

### 2.1. COFs with Different Chemical Structure Types

It is well known that covalent organic frameworks (COFs) are porous organic materials that are constructed by self-assembling materials linked together by covalent bonds [51,52]. Therefore, they possess unmatched biocompatibility and chemical stability, as well as high surface areas, high porosities, and ease of functionalization, similar to metal–organic frameworks (MOFs) and zeolitic imidazolate frameworks (ZIFs). The highly ordered π-π conjugated system in COFs and their independently accessible regular pores provide high levels of electronic conductivity. It is for this reason that these materials are often used as excellent photocatalysts, for gas adsorption and separation, electrochemical sensing, and energy storage applications [53,54]. The following subsections provide an overview of the different chemical structures of COFs.

#### 2.1.1. The B-O Structure of COFs

**Figure 2:**
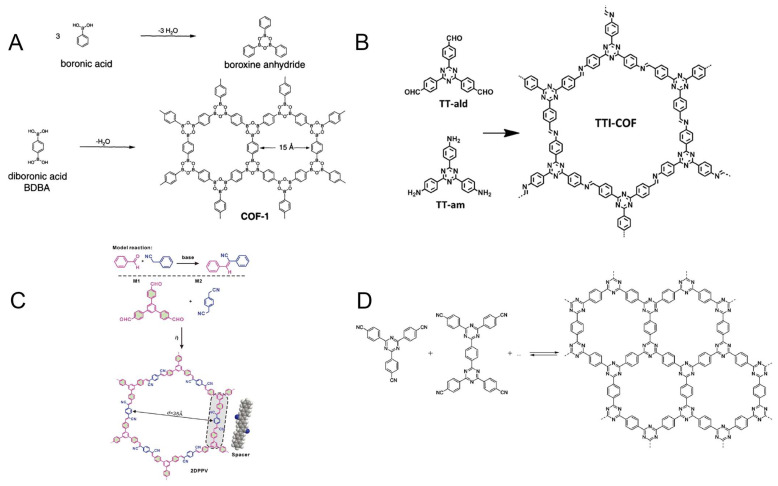
Schematic representation of COF materials synthesized with different chemical structures: (**A**) the B-O structure, Ref. [47], Copyright 2005, The American Association for the Advancement of Science; (**B**) the imine structure, Ref. [56], Copyright 2016, John Wiley and Sons; (**C**) the C=C structure, Ref. [60], Copyright 2016, The Royal Society of Chemistry; and (**D**) the triazine structure, Ref. [61], Copyright 2009, American Chemical Society.

In 2005, Yaghi et al. synthesized COF-1 and COF-5, typical examples of the B-O structure [47] (Figure 2A). One method for the synthesis of COF-1 is based on the self-condensation of phenylboronic acid, a process in which the boronic acid molecules in phenylboronic acid undergo dehydration in order to form a two-dimensional B_3_O_3_ ring (boroxine ring). The boronic acid molecules in 1,4-phenyldiboronic acid have the same capability of undergoing condensation during dehydration to form a layered hexagonal framework (COF-1). It is possible to obtain an extendable layered structure (COF-5) by dehydrating and condensing 1,4-phenyldiboronic acid with 2,3,6,7,10,11-hexahydroxytriphenylene. However, it should be noted that these types of COFs have a poor water stability due to the reversible reaction of boronic acid ester formation, which causes their hydrolysis when exposed to acid, alkali, or atmospheric water vapor, thus impairing the quality of their framework [55].

#### 2.1.2. The Imine Structure of COFs

COFs are typically connected by imine bonds when amines and aldehydes undergo the Schiff base reaction, which produce C=N bonds [56] (Figure 2B). Yaghi et al., in 2009, reported the first example of this type of COF. During the experimental process, COF-300 was found to be structurally stable at 490 °C and insoluble in both water and common organic solvents [57]. The application prospects of COFs connected by imine bonds are greater than those of COFs linked by B-O bonds. In addition, COFs with oxime bonds can be considered as another type of imine-based COFs, which are formed by reacting hydrazine compounds with aldehydes or ketones, such as COF-42 and COF-43 [58].

The Banerjee group introduced functional groups -OH into their structures in order to improve their stability and crystallinity. As a result of the reaction between 2,5-dimethoxybenzaldehyde (Dma) and 2,5-dihydroxybenzaldehyde (Dha) with 5,10,15,20-tetra(4-aminophenyl)-21H,23H-porphine (Tph), the COFs of interest were synthesized. The results indicated that, compared to DmaTph, the O-H···N=C interaction in the DhaTph structure partially protected the COFs from hydrolysis under aqueous and acidic conditions, thereby improving their crystallinity and porosity [59].

#### 2.1.3. The C=C Structure of COFs

During the Knoevenagel condensation reaction, the active methylene group in a compound is dehydrated and condensed with an aldehyde or ketone under the catalysis of a base, leading to the formation of a thermally stable compound. In spite of this, due to the limitations of the reaction conditions, it has been difficult to apply this principle to the synthesis of COFs for a long time. The first time this principle was applied was in 2016, when Zhang and Feng synthesized two-dimensional conjugated COFs (2DPPV) with C=C connectivity [60]. Figure 2C illustrates the reaction process, in which diphenyl dinitrile and 1,3,5-tris(4-formylphenyl)benzene are used as monomers to produce a two-dimensional-layered framework. By activating it further, carbon nanosheets can be formed, increasing their surface area from 472 m^2^ g^−1^ to 880 m^2^ g^−1^. In the field of electrochemistry, it has been successfully applied as a capacitor and catalyst, demonstrating its great potential.

#### 2.1.4. The Triazine Structure of COFs

Covalent triazine-based frameworks (CTFs), which are triazine-based COFs, were first synthesized in 2008 by Kuhn et al. The self-polymerization of dinitrile occurs under the catalysis of zinc chloride at 400 °C, followed by the polymerization into polymers based on the triazine structure [61] (Figure 2D). It is important to note that these COFs have excellent thermal stability as well as chemical stability, but because of the high temperatures and strong acid catalysis required for the reaction process, their subsequent applications are limited. There are also many research groups that are dedicated to developing milder preparation methods.

#### 2.1.5. The Other Structure of COFs

Furthermore, there are many other ways of connecting COFs besides those mentioned above, such as C-C [62], aminal [63], imide [64], ester [65], and quinoline [66]. Their chemical stabilities, thermal stabilities, large surface areas, and designable pore sizes make them highly promising in a wide range of applications.

Due to their high functionality, as well as their highly ordered π-π conjugated systems, independent open pores, and high specific surface area, COF materials facilitate rapid electron transfer and energy storage. Moreover, COFs possess electrodes with high specific surface areas and a dense exposure of catalytic active sites, and the interconnected pores facilitate diffusion and contact between the analytes and the active sites. Therefore, it has been found that electrodes constructed using COFs directly or with electrochemically active molecules are ideal electrodes for electrochemical sensing analysis [22,67]. It has been reported that various types of electrochemically active COFs have been developed as a result of COFs’ ability to be easily controlled by functional groups. In Wang’s research team, an electrochemically active two-dimensional COF_Thi-TFPB_ was synthesized by introducing sulfur as an electroactive monomer, which was then grown on carbon nanotube surfaces functionalized with amines. It was applied to the construction of ascorbic acid (AA) and pH sensors [68]. In Lu’s research laboratory, a topological skeleton COF-LZU1 based on Fe^3+^ coordination was prepared, as well as Fe_3_O_4_/N composites for enzyme-free plasma component detection [69]. According to Wang’s research group, the self-redox-active COF_DHTA-TTA_ was used as an electroactive material in the construction of electrochemical sensors for H_2_O_2_, pH, glucose, etc., which demonstrated excellent stability and performance in the detection of these targets [70]. Zhang’s research group utilized COF nanocomposites doped with Au NPs as signal probes for catechin testing [71]. It is important to recognize that the output of electrical signals is a critical component in the design of electrochemical sensors. It follows that the crucial issue in the application of COFs to electrochemical sensing is the development of more versatile electrode materials, the design of electroactive COFs, or the development of COFs that are capable of performing more than one function. Consequently, COF materials may be developed and applied to electrochemical analysis with some potential and feasibility.

### 2.2. Principles of Electrochemical Detection and COF-Based Electrochemical Sensors

The electrochemical sensor detects and quantifies chemical components in a sample using electrochemical principles. The selection of electrode materials is essential for the construction of the electrochemical sensing interface, and COFs have gained considerable attention as highly promising electrode materials. It is well known that COFs possess a variety of porous structures, low toxicity, and excellent biocompatibility, which make them ideal for the construction of sensing interfaces. The application of COFs to electrochemical sensors is therefore becoming increasingly popular. There have been more than 100 publications in this field (from WOS) over the last five years.

It is also possible to modify COFs with different functional groups or metal ions to develop a number of highly specific and targeted sensors [72]. In addition to their outstanding stability, they are widely used in electrochemical sensors due to their high durability [73]. With electrochemical sensors based on COFs, the real-time monitoring of analytes is possible with minimal sample preparation and rapid analysis. Over the last several years, COFs have attracted an increasing amount of attention owing to their excellent performance and their ability to be used in the development of new electrochemical sensors. There has been a steady increase in the number of articles related to COF-based electrochemical sensors since Wang and colleagues detected Pb^2+^ using COF-based electrochemical sensors in 2018 [74]. It is primarily in the food industry that electrochemical sensors based on COF are used for the detection of hazards associated with food.

## 3. Recent Advances in COF-Based Electrochemical Sensors for Food Safety Analysis

**Figure 3:**
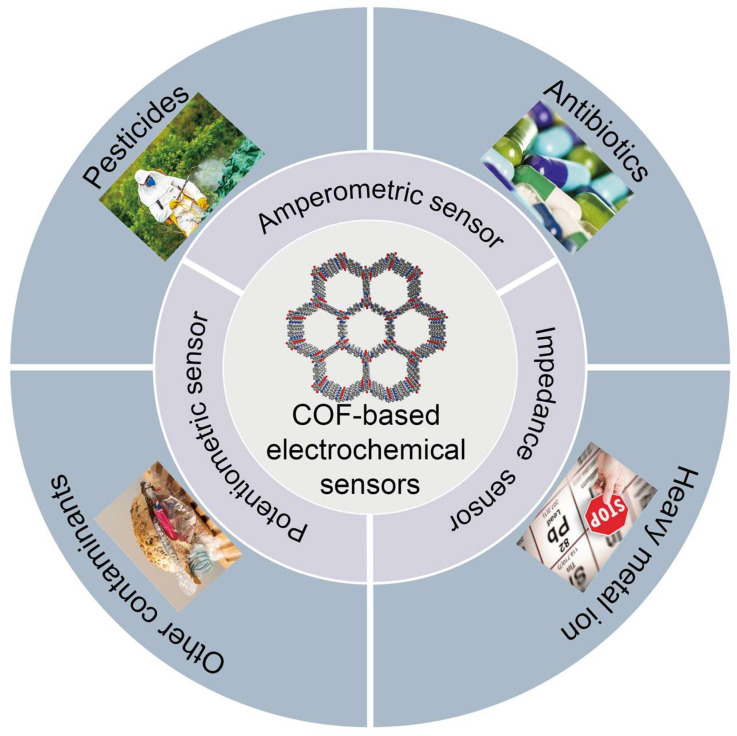
COF-based electrochemical sensing platforms in food safety applications.

In the field of electrochemical sensing, COFs have been successfully applied due to their captivating structure and properties [73]. It is currently possible to detect a wide range of food contaminants using electrochemical sensors based on COFs. COFs are advantageous because they provides a large number of binding sites and π-π stacking interactions, which speed up charge transfer and enhance the electrochemical performance of the sensor [75]. Consequently, they exhibit a high degree of selectivity, a high sensitivity, and a rapid response time. Throughout this review, COF-based electrochemical sensors are presented and discussed as a means of detecting various food hazards, including pesticides, heavy metal ions, antibiotics, and other relevant substances (Figure 3 and Table 1).

### 3.1. Detection of Pesticides

Pesticides play an important role in agricultural production; however, the residues they leave behind and their degradation products can pose serious threats to ecosystems and human health. It is possible for them to disrupt the ecological balance as well as the major functions of the human body, including the immune system, nervous system, and endocrine system, which can lead to a variety of diseases. There has been a growing concern regarding rapid detection techniques for detecting pesticides in a timely and accurate manner.

**Figure 4:**
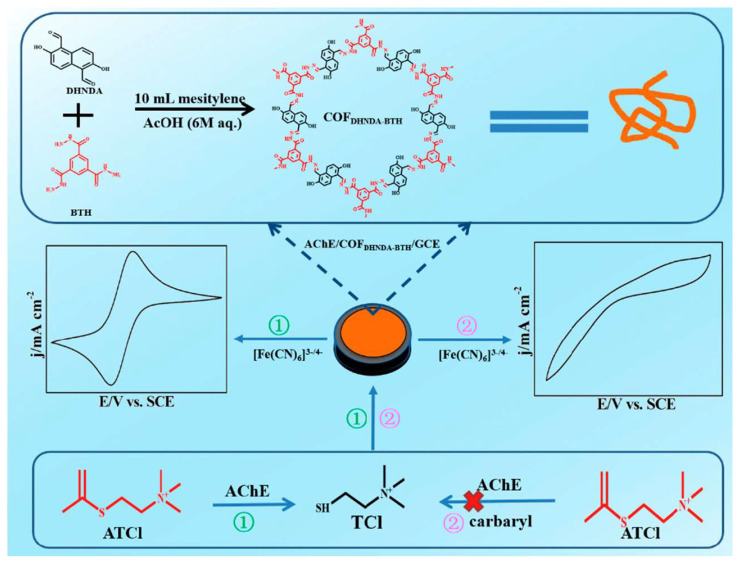
Process of preparing and utilizing the electrochemical sensor for AChE/COF_DHNDA-BTH_, Ref. [77], Copyright 2022, MDPI.

It has been reported that some pesticides inhibit enzymes that catalyze substrates, resulting in changes in signal levels and the indirect measurements of pesticide levels [76]. As a consequence of this approach, factors such as enzyme loading and activity have a considerable impact on the performance of the sensor. An ideal platform for enzyme immobilization and protection of enzyme activity is COF, with its large specific surface area and adjustable pore size. Through an amine–aldehyde condensation reaction, Chen et al. constituted a COF that is rich in C=O, NH, and OH groups [77]. The biocatalytic activity of acetylcholinesterase was greatly enhanced when it was immobilized on paper electrodes with COF (Figure 4). This biosensor had a linear range of 0.48–35 μmol/L, with a limit of detection (LOD) of 0.16 μmol/L. Using this electrochemical biosensor, sevin from lettuce juice samples has also been detected. Furthermore, Wang et al. developed an electrochemical sensor for the detection of O,O-dimethyl-O-2,2-dichloroethenyl phosphorothioate (DDVP) by modifying an electrode with ethylene-based electroactive COF_Tab-Dva_ nanofibers (as carriers and conductors) [78]. By interacting with the ethylene groups in COF and the thiol groups in choline thiocholine, the ethylene groups in COF appear to be enriched on the electrode surface, thus improving the sensitivity of the electrode. The current response of the probe is altered due to the formation of repulsion with positively charged choline thiocholine and [Ru(bpy)_3_]^2+^. As a result, a low-potential pesticide detection can be achieved. Due to the reduction in the amount of thiol choline catalyzed by AChE as the concentration of pesticides increases, choline thiocholine is less repelled by [Ru(bpy)_3_]^2+^, resulting in the generation of redox current signals at the electrode surface. Providing enzymes with a microenvironment of superior chemical stability ensures that they will maintain a higher level of activity regardless of adverse external conditions. The research group of Lu [79] also synthesized COF that contained a large amount of carbonyl groups and used it for the construction of an electrochemical sensor capable of detecting para-hydroxybenzoate in cucumber samples. In addition, Song [80] and Wang [81] independently constructed electrochemical sensors to detect malathion and diazinon on the basis of COFs.

It is noteworthy that, despite the fact that there are few reports regarding the application of COF-based electrochemical sensors for the determination of pesticide residues, these reports demonstrate the promise of these sensors as pesticide analysis tools in the future. For these reasons, more research is urgently required in order to expand the types of COF composites and pesticides that can be evaluated.

### 3.2. Detection of Heavy Metal Ions

Heavy metal ions, such as mercury, lead, and cadmium, are widespread pollutants found in food. In recent years, heavy metal pollution has become a serious food safety concern due to the development of industries. Through numerous pathways in the food chain, these metals can enter the human body, causing chronic poisoning, neurological disorders, and even cancer [82]. Therefore, ensuring the safety of foods requires the detection of heavy metals. Although various analytical methods have been applied for the qualitative and quantitative analysis of heavy metals, such as atomic fluorescence spectroscopy [83], atomic absorption spectroscopy [84], and inductively coupled plasma mass spectrometry [85], these methods are often complex and expensive, thus limiting their application scope. It is therefore essential to continue to focus on developing rapid and sensitive detection methods.

Zhu and colleagues prepared a highly crystalline COF through the Schiff base reaction between triazine trinitrile (TPA) and 2,4,6-triformylphloroglucinol (TDBA) [86]. The result was the development of an electrochemical sensor based on COF_TDBA-TPA_ capable of simultaneously detecting Cd^2+^, Cu^2+^, Pb^2+^, Hg^2+^, and Zn^2+^ in drinking water. It was found that Cd^2+^, Cu^2+^, Pb^2+^, Hg^2+^, and Zn^2+^ had detection limits of 0.922 nM, 0.450 nM, 0.309 nM, 0.208 nM, and 0.526 nM, respectively. Furthermore, COF_TDBA-TPA_ was found to be capable of adsorbing Cd^2+^, Pb^2+^, Cu^2+^, and Hg^2+^. Using the Schiff base reaction of 2,5,8-triamino-s-heptazine (MELE) and 4,4’-(phenyl-(c)[1,2,5]-thiadiazol-4,7-diyl)bisbenzaldehyde (BTDD), they synthesized a COF with multiple metal ion adsorption sites [87]. A COF_MELE-BTDD_ experiment was performed to detect Cd^2+^, Pb^2+^, Cu^2+^, and Hg^2+^, with LODs of 0.00474 μM, 0.00123 μM, 0.00114 μM, and 0.00107 μM, respectively.

**Figure 5:**
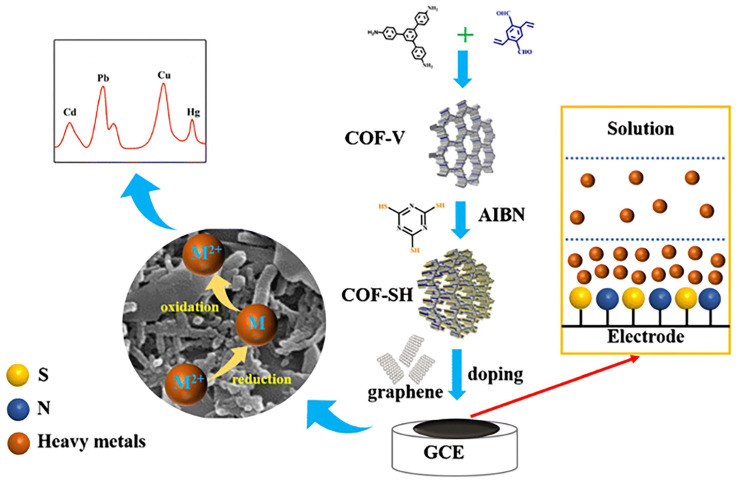
Process of preparing and utilizing the electrochemical sensor for G/COF-SH/GCE, Ref. [72], Copyright 2021, Springer Nature.

A triazine-COF modified glassy carbon electrode based on Madrakian’s and colleagues’ work has been developed as a novel, simple, sensitive, and fast electrochemical sensor for the simultaneous detection of Pb^2+^ and Hg^2+^ [88]. In terms of Pb^2+^ and Hg^2+^, the linear range was 0.01–0.3 μmol/L, and their lowest detectable limits were 0.72 × 10^−3^ and 1.2 × 10^−2^ μmol/L, respectively. Moreover, the detection of Pb^2+^ and Hg^2+^ in food samples was conducted using an electrochemical sensor. A novel glassy carbon electrode was proposed by Madrakian et al. in which a bismuth film, triazine-COF nano-composite materials, and Fe_3_O_4_ nanoparticles were incorporated into the electrode [89]. Glassy carbon electrodes were capable of selectively detecting Pb^2+^ with a limit of detection (LOD) of 0.95 nmol/L. Based on intercalated composite materials, Zhu and his colleagues developed an electrochemical sensor for the detection of heavy metal ions. Based on the scheme presented in Figure 5, COF-V was synthesized by reacting 1,3,5-tris(4-aminophenyl) benzene with 2,5-divinylterephthalaldehyde [72]. The reaction of AIBN, trithiocyanuric acid, and COF-V resulted in the preparation of COF-SH. Graphene and COF-SH were intercalated onto a glassy carbon electrode (GCE) to produce the intercalated composites. As a result of the good enrichment effect of COF-SH on heavy metal ions and the superior conductivity of graphene, the electrochemical sensor demonstrated excellent performances in the detection of heavy metal ions. Cd^2+^, Pb^2+^, Cu^2+^, and Hg^2+^ each have a detection limit of 0.3, 0.2, 0.2, and 1.1 μg/L, respectively.

### 3.3. Detection of Antibiotics

It has been demonstrated that antibiotics are effective for inhibiting or killing pathogens; thus, they are widely used in the prevention and treatment of diseases caused by bacteria, fungi, molds, or other microorganisms [90]. Antibiotics have become more widely used in the livestock industry as a result of the continued expansion of the industry. It is becoming increasingly important to address the issue of antibiotic residues in this case. There are a number of health problems that can be caused by excessive residual antibiotics, including abnormal blood levels, liver toxicity, and allergic reactions [91]. Research has utilized COFs as scaffolds for immobilizing recognition elements or enhancing electrochemical performance using electroactive COFs for antibiotic detection using COF-based electrochemical sensors. There is no doubt that electrochemical sensors based on COF are highly sensitive, stable, and interference resistant. Nonetheless, the layered structure of 2D COFs allows for an easy encapsulation of their internal active sites, which may restrict the transfer of electrons. It is also essential to consider coating uniformity in order to ensure that modified electrodes perform as expected.

The use of quinolone antibiotics in the treatment and prevention of diseases in humans and animals is widespread. There may be negative environmental and health effects associated with the excessive use of antibiotics. As reported by Du et al., Py-M-COF is synthesized by the condensation reaction of 1,3,6,8-tetra(4-formylphenyl)pyrene (TFPPy) with cyanuric triamide [23]. The results of electrochemical impedance spectroscopy (ESI) measurements indicated that the Py-M-COF electrochemical sensor could detect enrofloxacin (ENR) and ampicillin (AMP) with extreme sensitivity. The linear response range was 0.12–2000 pg/mL for ENR and 0.001–1000 pg/mL for AMP, respectively, with the lowest detection limits of 6.07 fg/mL and 0.04 fg/mL. It was found that the COF-based sensing system had a higher sensitivity than graphitic carbon nitride (g-C_3_N_4_) and amino-functionalized graphene oxide (GO-NH_2_). COF contains a π-conjugated framework, which provides a higher charge carrier mobility for signals and additional anchoring points for aptamers. It has been demonstrated that Pan et al. prepared TAPB-PDA-COFs/AuNPs by in situ embedding of Au nanoparticles within TAPB-PDA-COFs (formed by the Schiff base reaction of 1,3,5-tri(4-aminophenyl)benzene (TAPB) and p-phenylenedialdehyde (PDA)) [92]. TAPB-PDA-COFs/AuNPs/GCE exhibited a high performance in ENR determination; the results demonstrated that ENR had two linear response ranges between 0.05–10 μM and 10–120 μM, with a LOD of 0.041 μM.

The COF@NH_2_-CNT composite material was prepared by Sun et al. [67]. to detect furazolidone (NF), by taking advantage of the high surface area of COFs and the excellent conductivity of NH_2_-CNTs. Due to COFs’ efficient adsorption capacity for furazolidone, the sensor was highly sensitive and responded rapidly. COFs were further applied to electrochemical sensors through this strategy [67]. Through the combination of COF_TFPB-DHzDS_, Pt NPs, and rGO, Du and his colleagues developed an electrochemical sensor for the sensitive determination of furazolidone [93]. TFPB and 2,5-bis(3-(ethylthio)propoxy)benzaldehyde hydrazone (DHzDS) were reacted via the Schiff base reaction to produce COF_TFPB-DHzDS_, which was then grown on the surface of rGO-NH2. An in situ reduction method was used to load the Pt NPs onto the COF_TFPB-DHzDS_@rGO. Despite the low detection limit of 0.23 μM and a wide linear range of 0.69 μM to 110 μM, the paper-based electrochemical sensor had a high level of sensitivity.

**Figure 6:**
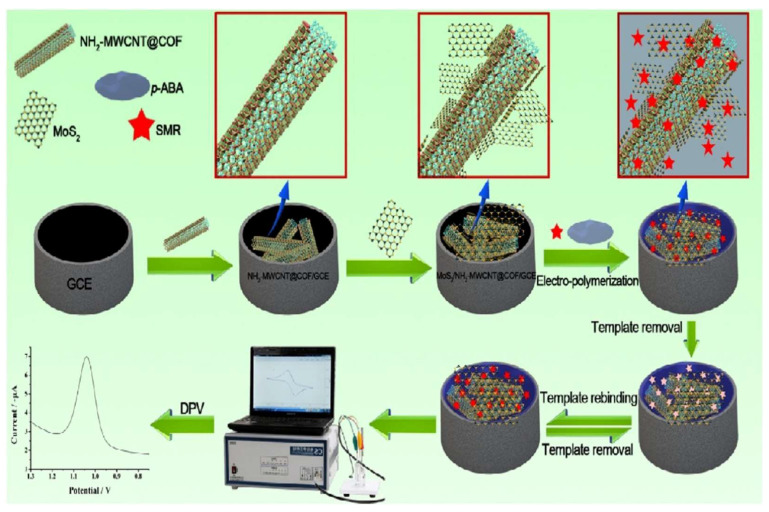
Process of preparing and utilizing the electrochemical sensor for MIP/MoS_2_/NH_2_-MWCNT@COF/GCE, Ref. [94], Copyright 2019, Elsevier B.V.

Antibiotics classified as sulfonamides are broad-spectrum antibiotics used exclusively for treating infections caused by bacteria. Xu et al. developed an electrochemical sensor that is capable of detecting sulfonamide drugs (SMRs) (Figure 6) [94]. MIP/MoS_2_/NH_2_-MWCNT@COF/GCE was produced by coating GCE with NH_2_-MWCNT@COF and MoS_2_ nanosheets, followed by electrochemical polymerization to obtain MIP/MoS_2_/NH_2_-MWCNT@COF/GCE. It was found that the electrochemical sensor prepared for SMR showed a broad response range, from 3.0 × 10^−7^ M to 2.0 × 10^−4^ M, with the lowest detection limit of 1.1 × 10^−7^ M.

It is commonly known that tetracycline (TC) is a type of antibiotic that can lead to drug resistance and other side effects, such as allergic reactions, kidney toxicity, and liver damage. The detection of tetracycline antibiotics is currently performed using electrochemical sensors based on COFs in order to further improve their stability and portability. A portable on-site electrochemical sensor similar to that proposed by Yukun Yang et al. [95] based on the use of surface molecularly imprinted polymers (MIPs) modified with magnetic COFs (Fe_3_O_4_@COFs@MIPs) for the sensitive and rapid determination of TC has been proposed. TC is detectable at concentrations from 1 × 10^−10^ to 1 × 10^−4^ g/mL, with a limit of detection (LOD) of 2.4 × 10^−11^ g/mL. Milk and chicken samples have also been successfully tested using the prepared sensor.

### 3.4. Detection of Other Contaminants

Other contaminants, such as illegal additives, are also threats to the safety of food, in addition to the previously mentioned pollutants. It is possible to selectively detect these targets through the design of COF-based sensing approaches in order to address these challenges.

There is no doubt that tertiary butylhydroquinone (TBHQ), a good antioxidant, plays a critical role in the prevention of lipid oxidation, but the high doses of TBHQ may cause carcinogenesis [96]. Using Co_3_O_4_@TAPBDMTP-COF as the sensor substrate, Chen et al. were able to more easily and rapidly detect TBHQ, owing to the excellent electrocatalytic property and the large surface area of COFs [97]. Compared with other methods, this approach exhibits higher sensitivity and selectivity towards TBHQ, with a limit of detection as low as 0.02 μM, and it can effectively detect the lower levels of TBHQ present in edible oil samples. There are many adverse effects associated with bisphenols, which are commonly found in plastic food packaging materials [98]. In their study of bisphenol BPS and bisphenol A, Qiao et al., developed a ratio electrochemical sensor able to measure both compounds simultaneously. As a result of the modification of carbon cloth electrodes with silver nanoparticles (COF/AgNPs/CC), this ratio sensor exhibits an excellent electrocatalytic activity toward both bisphenol A and bisphenol BPS, demonstrating a large amount of electrocatalytic surface area and good conductivity [99]. There is no difference between the detection limits for bisphenol A and bisphenol B at 0.15 μmol/L.

**Table 1:** Statistical analysis of the performance of electrochemical sensors based on different coefficients of friction.

Electrode	Target Substance	Detection Methods	LOD	Linear Range	References
AChE/COF_DHNDA-BTH_/GCE	Carbaryl	Cyclic voltammetry	0.16 μmol/L	0.48–35 μmol/L	[77]
AChE/COF_Tab-Dva_/GCE	DDVP	Cyclic voltammetry	0.11 μM	0.33–30 μM	[78]
GC/COF1/AChE/GCE	Paraoxon	Cyclic voltammetry	1.4 ng/mL	10–1000 ng/mL	[79]
AChE/COF-LZU1/3D-KSC	Trichlorfon	Differential pulse voltammetry	0.067 ng/mL	0.2–19 ng/mL	[80]
COF@MWCNTs	Malathion	Differential pulse voltammetry	0.5 nM	1–10 nM	[81]
COF_TDBA-TPA_/GCE	Cd^2+^	Square wave anodic stripping voltammetry	0.922 nM	2.8–8000 nM	[86]
	Pb^2+^		0.309 nM	0.939–4000 nM	
	Cu^2+^		0.45 nM	1.36–8000 nM	
	Hg^2+^		0.208 nM	0.632–8000 nM	
COF_MELE-BTDD_/GCE	Zn^2+^	Square wave anodic stripping voltammetry	0.526 nM	1.41–7000 nM	[87]
	Cd^2+^		4.74 nM	14.2–4000 nM	
	Pb^2+^		1.23 nM	3.7–4000 nM	
	Cu^2+^		1.14 nM	3.4–4000 nM	
	Hg^2+^		1.07 nM	3.2–4000 nM	
SNW1/GCE	Pb^2+^	Anodic stripping square wave voltammetry	0.00072 μmol/L	0.01–0.3 μmol/L	[88]
	Hg^2+^		0.01211 μmol/L	0.05–0.3 μmol/L	
Fe_3_O_4_@SNW1/GCE	Pb^2+^	Square wave anodic stripping voltammetry	0.95 × 10^−3^ μmol/L	0.003–0.3 μmol/L	[89]
G/COF-SH/GCE	Cd^2+^	Square wave voltammetry	0.3 μg/L	1–1000 μg/L	[72]
	Pb^2+^		0.2 μg/L	1–800 μg/L	
	Cu^2+^		0.2 μg/L	1–800 μg/L	
	Hg^2+^		0.1 μg/L	5–1000 μg/L	
AptENR/Py-M-COF/AE	Enrofloxacin	Electrochemical impedance spectroscopy	6.07 fg/mL	0.01−2000 pg/mL	[23]
AptAMP/Py-M-COF/AE	Ampicillin		0.04 fg/mL	0.001−1000 pg/mL	
TAPB-PDA-COFs/AuNPs/GCE	Enrofloxacin	Square wave anodic stripping voltammetry	0.041 μmol/L	0.05−10 μmol/L, 10−120 μmol/L	[92]
COF@NH_2_-CNT/GCE	Furazolidone	Differential pulse voltammetry	7.75 × 10^−8^ M	0.2–100 μM	[67]
PtNP/COF_TFPB-DHzDS_@rGO/ePAD	Furazolidone	Differential pulse voltammetry	0.23 μM	0.69−110 μM	[93]
MIP/MoS_2_/NH_2_-MWCNT@COF/GCE	Sulfamerazine	Differential pulse voltammetry	1.1 × 10^−7^ M	3.0 × 10^−7^−2.0 × 10^−4^ M	[94]
Fe_3_O_4_@COFs@MIPs/SPE	Tetracycline	Differential pulse voltammetry	2.4 × 10^−1^ g/mL	1 × 10^−10^–1× 10^−4^ g/mL	[95]
Co_3_O_4_@TAPBDMTP-COF	TBHQ	Differential pulse voltammetry	0.02 μM	0.05−1, 1–4 × 10^2^ μM	[97]
COF/AgNPs/CC	Bisphenol A	Differential pulse voltammetry	0.15 μmol/L	0.5–100 μmol/L	[99]
	Bisphenol S		0.15 μmol/L	0.5–100 μmol/L	

## 4. Conclusions

The purpose of this review has been to highlight the outstanding potential of COFs as emerging porous materials for electrochemical sensing, in particular to ensure food safety. It is becoming increasingly evident that COFs (covalent organic frameworks) are emerging porous materials with a high crystallinity, a good degree of stability, and a controllable pore size and topology. In the field of electrochemical sensing, COFs have exhibited great potential because of their flexible design. Analytes can undergo adsorption and electrochemical reactions facilitated by COFs due to their high surface area and porosity. It is possible to enhance the selectivity and sensitivity of COFs by introducing functional groups. Furthermore, the solid-state nature of COFs makes them easy to integrate with sensing platforms, enhancing their repeatability and stability.

Food safety can be improved through the use of electrochemical sensors, as they offer a high level of accuracy, simplicity, cost-effectiveness, and rapid response. COF-based electrochemical sensors are providing a platform for ensuring food safety and quality. Various designs and types of COFs have been reported, including B-O structures, imine bonds, C=C structures, triazine structures, and other types of connection. Further, the reports discuss methods for improving the performance of COF-based electrochemical sensors and discuss their application for the detection of food pollutants such as pesticides, heavy metal ions, antibiotics, etc.

In spite of the excellent performance and promising prospects of COF-based electrochemical sensors, they exhibit a few challenges, pertaining to enhanced stability and repeatability, miniaturization, and on-site operation. In spite of these challenges, COF-based electrochemical sensors continue to hold great promise in the field of food analysis. It is anticipated that future research will emphasize the combination of novel COFs and advanced electrochemical technologies in order to provide electrochemical sensors with excellent analytical performance.

As a part of the design of COFs, the appropriate pore size and the specific functional groups will be considered based on the physical and chemical properties of the target analytes, which will enhance the selectivity of the modified electrode. It has been proposed that post-modification methods could be employed in order to modify the prepared COF materials and achieve improved properties. Cui et al. modified the COFs with cyclodextrin in order to enhance their ability to transport amino acids selectively [100]. It was found in the study of Li et al. that carboxylic acid groups added to COF materials significantly enhanced the adsorption of heavy metals such as cadmium and mercury [101]. Consequently, the modification of COF materials will enhance their application in the electrochemical detection industry in the future. Moreover, the post-synthetic modifications and alternative methods of enhancing the stability of COFs, particularly imine-based COFs, will increase the requirement for recycling. Additionally, developing mild and effective methods for the modification of COFs on electrodes is crucial in order to ensure optimal contact and minimize any adverse effects on the efficiency of the detection process.

Electrochemical sensors based on COFs are typically constructed by coating or modifying conventional electrodes, but the direct integration of COFs into the device structure can further enhance their sensing capabilities. The advantage of this approach is that it minimizes the signal-to-noise ratio and increases the surface area for the optimum binding and reaction of analytes. It is also advantageous to integrate multiple COF-based sensors on a single substrate, which allows for multiplexed detection, thus increasing the overall efficiency of the system.

COF-based electrodes have recently been shown to have potential applications in the analysis of biomedical and environmental pollutants. It has been demonstrated, for example, that COF-modified electrodes exhibit superior sensitivity, selectivity, stability, and reproducibility when compared with classical electrodes. In particular, COF-modified electrodes are remarkably recyclable even after multiple cycles, demonstrating their durability. While COFs have made significant advancements in electroanalytical chemistry, their application is still relatively new, and challenges must be overcome in order to develop COF-based electrochemical sensors in the future.

Looking ahead, it is vital to develop the reasonable design and controllable synthesis of multifunctional electrode materials based on COFs in order to improve their selectivity towards food pollutants and to enhance their stability and repeatability while achieving the miniaturization of the sensors. Moreover, the biocompatibility study of COF-based electrochemical sensors should be strengthened in order to expand their applications in the detection of biological food contaminants. Additionally, it would be beneficial to address the issue of electrode surface fouling and extend the lifetime of sensors by designing and synthesizing COF electrode materials with anti-fouling properties.

Electrochemical sensors for COFs will be developed in part by the advancement of synthetic techniques and the development of analytical chemistry, among other factors. With the development of new synthetic techniques, COFs with more stable and controllable structures can be synthesized, enabling further investigation into the potential applications of COFs in areas such as food safety and biology. Analytical chemistry advancements have contributed to improving the performance and functionality of sensors. Since the development of synthetic techniques for COFs, researchers have been able to synthesize COFs with more stable and controlled structures. Recently, a number of new synthetic strategies and methods have been developed, including solvent evaporation, cosolvent synthesis, and interfacial synthesis. Electrochemical sensors for COFs can now be developed using these new techniques, providing more options and possibilities. Electrochemical sensors for COFs must also take into account a number of analytical chemistry considerations. It may be necessary, for example, to select the correct sensor materials and synthesis methods in order to achieve a higher level of sensitivity and selectivity. It is also important to pay attention to performance indicators, including stability, repeatability, and practicality. A further enhancement of the performance and functionality of the sensors can be achieved through the interaction of COFs with other functionalized materials. The development of electrochemical sensors with COFs in the future will be driven by these trends, which will lead to wider applications and an increased market potential.

In summary, COF-based electrochemical sensors are still in the early stages of application in the field of food analysis, but this innovative research field holds great promise. It has been possible to detect antimicrobials, pesticides, and heavy metals using some of these techniques. It is anticipated that continued research into the design and synthesis of COFs, in conjunction with advanced electrochemical technologies, will contribute to the enhancement of the analytical performance of electrochemical sensors and the development of food safety testing systems.

## Data Availability

The data used to support the findings of this study is contained within the article.
